# Supplementary material for: Fast and effective molecular property prediction with transferability map
Source: Commun Chem. 2024 Apr 17;7:85. doi: 10.1038/s42004-024-01169-4 (PMC11024153; doi:10.1038/s42004-024-01169-4)
Supplement: Supplementary file 2 — Supplementary Information [file 42004_2024_1169_MOESM2_ESM.pdf]

Supplementary Information

Supplementary Note1 - Details of PGM algorithm

The computation process of PGM based transferability quantification is shown in Algorithm 1.

Algorithm 1 Principal Gradient-based Measurement

Input: Model $F(g, w)$ initialized from (g_0, w_0), source datasets $\{(D_{s(n)}\}_{n=1}^{N}$, target dataset D_t

Output: Transferability from source tasks $\{(T_{s(n)}\}_{n=1}^{N}$ to target task T_t

```
for D ∈ {(D_s(n), D_t}_{n=1}^N do                          ▷ PGM
    for epoch = 1, ..., K do
        Calculate principal gradient PGM(g_0) by Eqn. (1)
    end for
end for
for n in N do                                              ▷ Transferability quantification
    Calculate transferability PGM_{T_s(n)→T_t}(g) by Eqn. (7)
end for
```

Supplementary Note2 - Detailed description of datasets

Table S1 provides an overview of the molecular datasets used in our paper from MoleculeNet[1]. They are categorized into physiology, biophysics, and physical chemistry. The detailed information for the datasets is listed as follows:

Biophysics BACE[2] has been established to gather compounds that could act as the inhibitors of human β-secretase 1 (BACE-1). HIV[3] is from the Drug Therapeutics Program (DTP) AIDS Antiviral Screen, and it aims at predicting inhibit HIV replication. PCBA[4] is a dataset that comprises the biological activities of small molecules generated through high-throughput screening methodologies. Maximum Unbiased Validation (MUV)[5] is another sub-database from PCBA, and is obtained by applying a refined nearest neighbor analysis.

Table S1 Summary of all the benchmarks for molecular property predictions used in this work.

Task Type	Metric	Category	Dataset	# Tasks	# Compounds
Classification	ROC-AUC	Biophysics	BACE	1	1,513
			HIV	1	41,127
			MUV	17	93,087
			PCBA	128	437,997
		Physiology	BBBP	1	2,039
			ClinTox	2	1,478
			SIDER	27	1,427
			Tox21	12	7,831
			ToxCast	617	8,575
Regression	RMSE	Physical chemistry	ESOL	1	1,128
			FreeSolv	1	642
			Lipophilicity	1	4,200

Physiology The Blood-Brain Barrier Penetration (BBBP)[6] dataset measures whether a molecule will penetrate the central nervous system. All three datasets, Tox21[7], ToxCast[8], and ClinTox[9] are related to the toxicity of molecular compounds. The Side Effect Resource (SIDER)[10] dataset stores the adverse drug reactions on a marketed drug database.
Physical Chemistry ESOL[11] measures aqueous solubility of common organic small molecules. FreeSolv[12] measures hydration free energy of small molecules in water, which is obtained through molecular dynamics simulations. Lipophilicity is a subset of ChEMBL[13] measuring the molecule octanol/water distribution coefficient.

Supplementary Results

Table S2 details the time for transferability quantification (including the time for computing principal gradient at 1, 10, and 20 epochs, as well as the time for computing PGM distance) and the time for fine-tuning. Notably, fine-tuning demands 3 to 16 times more time than transferability quantification for an equivalent 20-epoch period. Furthermore, the transferability quantification outcomes can serve as a reference standard for transfer learning across these datasets, negating the need for repeated recalculations. In contrast, fine-tuning requires re-computation for different scenarios. This highlights the computational efficiency of PGM.

Table S2 Training time (seconds) for different target datasets, utilizing the remaining 11 datasets as sources for each target.

Target Dataset	BACE	Tox21	ESOL
PGM (1epoch)		3.94	
PGM (10epoch)		26.28	
PGM (20epoch)		50.63	
PGM distance	1.58	1.65	1.54
fine-tuning (20epoch)	248.81	825.52	160.16

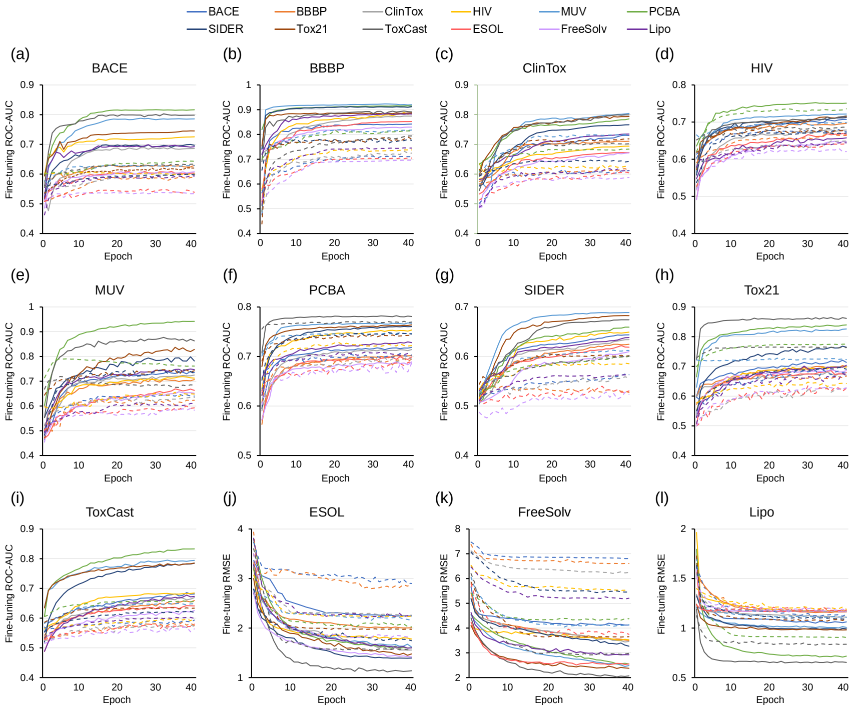

Fig. S1 Training and validation curves of fine-tuning on the 12 target datasets. The 12 targets include 9 classification datasets (**a**∼**i**) and 3 regression datasets (**j**∼**l**). The solid and dashed lines indicate the training and validation curves, respectively.

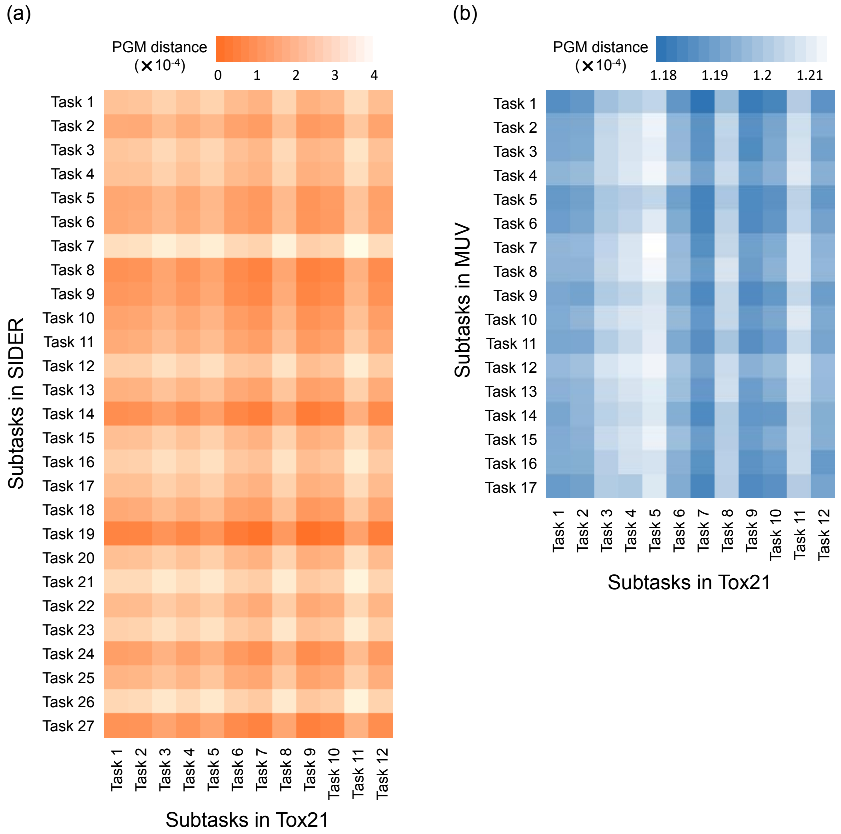

Fig. S2 Transferability maps generalizable across subtasks within molecular properties. a Transferability map illustrating the task-relatedness between two different physiology multitask datasets: Tox21 and SIDER. **b** Transferability map illustrating the task-relatedness between one biophysics and one physiology multitask datasets: Tox21 and MUV.

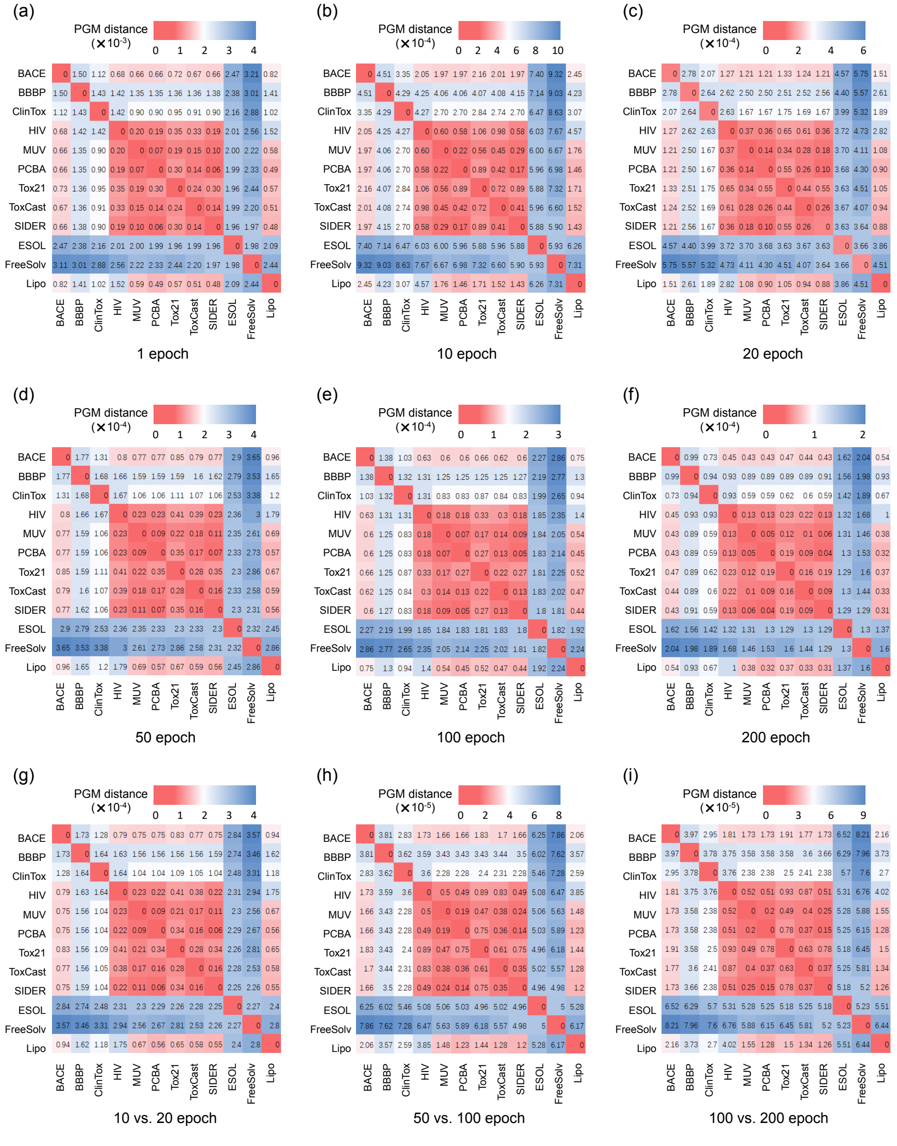

Fig. S3 Representative transferability maps and difference heatmaps. Transferability maps of performing PGM across epochs **a** 1, **b** 10, **c** 20, **d** 50, **e** 100, and **f** 200, and difference heatmaps for selected epoch intervals **g** 10 vs. 20, **h** 50 vs. 100, and **i** 100 vs. 200. Red cells with smaller values indicate closer PGM distances, while blue ones with larger values indicate longer PGM distances.

Supplementary References

[1] Wu, Z. *et al.* Moleculenet: a benchmark for molecular machine learning. *Chemical science* **9** (2), 513–530 (2018).

[2] Subramanian, G., Ramsundar, B., Pande, V. & Denny, R. A. Computational modeling of β-secretase 1 (bace-1) inhibitors using ligand based approaches. *Journal of chemical information and modeling* **56** (10), 1936–1949 (2016).

[3] Riesen, K. & Bunke, H. Iam graph database repository for graph based pattern recognition and machine learning. In *Structural, Syntactic, and Statistical Pattern Recognition: Joint IAPR International Workshop, SSPR & SPR 2008, Orlando, USA, December 4-6, 2008. Proceedings* 287–297 (Springer, 2008).

[4] Wang, Y. *et al.* Pubchem's bioassay database. *Nucleic acids research* **40** (D1), D400–D412 (2012).

[5] Rohrer, S. G. & Baumann, K. Maximum unbiased validation (muv) data sets for virtual screening based on pubchem bioactivity data. *Journal of chemical information and modeling* **49** (2), 169–184 (2009).

[6] Martins, I. F., Teixeira, A. L., Pinheiro, L. & Falcao, A. O. A bayesian approach to in silico blood-brain barrier penetration modeling. *Journal of chemical information and modeling* **52** (6), 1686–1697 (2012).

[7] Hartung, T. Toxicology for the twenty-first century. *Nature* **460** (7252), 208–212 (2009).

[8] Richard, A. M. *et al.* Toxcast chemical landscape: paving the road to 21st century toxicology. *Chemical research in toxicology* **29** (8), 1225–1251 (2016).

[9] Gayvert, K. M., Madhukar, N. S. & Elemento, O. A data-driven approach to predicting successes and failures of clinical trials. *Cell chemical biology* **23** (10), 1294–1301 (2016).

[10] Kuhn, M., Letunic, I., Jensen, L. J. & Bork, P. The sider database of drugs and side effects. *Nucleic acids research* **44** (D1), D1075–D1079 (2016).

[11] Delaney, J. S. Esol: estimating aqueous solubility directly from molecular structure. *Journal of chemical information and computer sciences* **44** (3), 1000–1005 (2004).

[12] Mobley, D. L. & Guthrie, J. P. Freesolv: a database of experimental and calculated hydration free energies, with input files. *Journal of computer-aided molecular design* **28**, 711–720 (2014).

[13] Gaulton, A. *et al.* Chembl: a large-scale bioactivity database for drug discovery. *Nucleic acids research* **40** (D1), D1100–D1107 (2012).
